# Robust pathway-based multi-omics data integration using directed random walks for survival prediction in multiple cancer studies

**DOI:** 10.1186/s13062-019-0239-8

**Published:** 2019-04-29

**Authors:** So Yeon Kim, Hyun-Hwan Jeong, Jaesik Kim, Jeong-Hyeon Moon, Kyung-Ah Sohn

**Affiliations:** 1 Department of Computer Engineering, Ajou University, Suwon, 16499 South Korea; 2 Department of Molecular and Human Genetics, Baylor College of Medicine, Houston, TX 77030 USA; 3 Jan and Dan Duncan Neurological Research Institute, Texas Children’s Hospital, Houston, TX 77030 USA

**Keywords:** Multi-omics, Integrative analysis, Random walk, Pathway-based analysis, Breast cancer, Neuroblastoma

## Abstract

**Background:**

Integrating the rich information from multi-omics data has been a popular approach to survival prediction and bio-marker identification for several cancer studies. To facilitate the integrative analysis of multiple genomic profiles, several studies have suggested utilizing pathway information rather than using individual genomic profiles.

**Methods:**

We have recently proposed an integrative directed random walk-based method utilizing pathway information (iDRW) for more robust and effective genomic feature extraction. In this study, we applied iDRW to multiple genomic profiles for two different cancers, and designed a directed gene-gene graph which reflects the interaction between gene expression and copy number data. In the experiments, the performances of the iDRW method and four state-of-the-art pathway-based methods were compared using a survival prediction model which classifies samples into two survival groups.

**Results:**

The results show that the integrative analysis guided by pathway information not only improves prediction performance, but also provides better biological insights into the top pathways and genes prioritized by the model in both the neuroblastoma and the breast cancer datasets. The pathways and genes selected by the iDRW method were shown to be related to the corresponding cancers.

**Conclusions:**

In this study, we demonstrated the effectiveness of a directed random walk-based multi-omics data integration method applied to gene expression and copy number data for both breast cancer and neuroblastoma datasets. We revamped a directed gene-gene graph considering the impact of copy number variation on gene expression and redefined the weight initialization and gene-scoring method. The benchmark result for iDRW with four pathway-based methods demonstrated that the iDRW method improved survival prediction performance and jointly identified cancer-related pathways and genes for two different cancer datasets.

**Reviewers:**

This article was reviewed by Helena Molina-Abril and Marta Hidalgo.

## Background

For a better understanding of the biological basis of cancer and precise prediction of survival for cancer patients, integrative analysis of multi-omics data has been addressed in many studies [1–3]. Most integrative approaches used in cancer studies have focused on integrating multiple types of genomic data rather than using single omics profile. The use of multi-omics data has been valuable in its application to many different cancer types and it is necessary to reveal the underlying complex nature of biological mechanisms by analyzing human genomes at multiple genomic levels. To effectively combine different levels of omics data, several studies have led to the development of novel multi-omics data integration algorithms in order to predict phenotypic outcomes precisely and to discover biologically meaningful information [4–11]. Among recently proposed data integration methods, we focused primarily on network-based methods which can incorporate interactions between genes. Most network-based methods have focused on incorporating pathway or subtype information rather than using individual genomic features in different types of cancer datasets [9–18]. In this respect, pathway-based methods have been proposed for the identification of important genes within pathways.

To incorporate pathway information, Guo et al. [16] computed two summary measures to capture the pathway activity: the arithmetic mean and the median of the gene expression values of pathway member genes. They achieved better cancer classification performance and improved biological interpretability. Lee et al. [12] proposed a disease classification method based on pathway activities inferred for each patient. For each pathway, these authors summarized activity levels with condition-responsive genes (the pathway member genes whose combined expression show optimal discriminative power for the disease phenotype) by combining normalized z-transformed scores of genes (z-score method). A pathway level analysis of gene expression (PLAGE) measures the pathway activity profiles of a set of genes in each pathway, which are derived from a vector of the singular value decomposition of the given gene set [14]. PLAGE identified several biologically meaningful pathways using gene expression data from a study of type 2 diabetes and the effects of smoking on airway epithelia. Other pathway activity inference approaches have been proposed based on probabilistic inference for better cancer classification [13, 15, 17, 18]. PLAGE and the z-score method incorporate pathway information and transformed single genomic profiles into pathway profiles. However, they simply consider a pathway as a set of genes, and interactions between genes are not considered. Some pathway-based methods utilizing gene signatures or topological information utilizing gene interactions on a gene-gene graph have been studied.

A denoising algorithm based on relevance network topology (DART) integrates existing pathways with gene expression data by deriving perturbation signatures which reflect gene contributions in each pathway to obtain reliable molecular pathway activity predictions [10]. This work also showed that the encoded hub genes in expression correlation networks represent reliable markers of pathway activity in clinical tumor specimens. To consider the topological importance of the genes in the pathways that can be highly associated with diseases, Liu et al. [9, 11] proposed a directed random walk (DRW)-based pathway inference method to identify topologically important genes and pathways by weighting the genes in a gene-gene network. Although the DRW method only used gene expression data, this approach has also been applied to the integration of gene expression and metabolite data on a gene-metabolite graph, guided by pathway information [9]. However, those existing pathway-based methods, including DART and DRW, have limitations in that they only target a single genomic profile, generally obtained from gene expression data. In order to reflect the combined effect of different types of genomic profiles, we have previously proposed an integrative pathway-based method as an extension of the DRW method for multi-omics data (iDRW) [6]. In our previous research, we constructed an integrated gene-gene graph using gene expression and methylation profiles, and showed that the proposed method improved the survival prediction performance for breast cancer patients. We also showed that joint analysis of the methylation features and gene expression profiles can identify breast cancer-specific pathways.

One limitation of the iDRW method lies in the lack of analysis of other types of genomic profiles for different cancer studies. In the iDRW method, the gene expression and methylation data of breast cancer patients were studied. As copy number variants (CNVs) have shown a significant impact on gene expression [19], an understanding of the influence of CNVs on gene expression and clinical phenotypes in humans can contribute to a better understanding of disease. In this study, we investigated the impact of CNVs on gene expression for two different cancer types: breast cancer and neuroblastoma, utilizing the iDRW method.

**Fig. 1:**
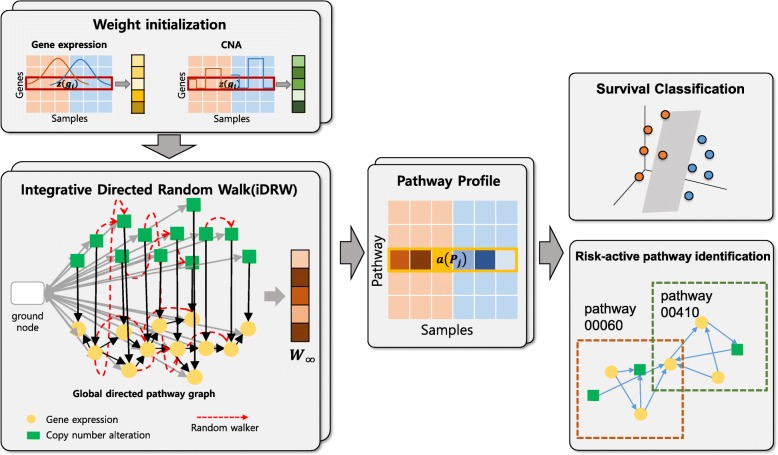
Overview of the proposed pathway-based multi-omics integration method for survival prediction

The main contributions of this study are as follows. First, we revamped a directed gene-gene interaction graph which reflects the interaction between gene expression and copy number alteration. Considering different data distributions of gene expression and copy number data, we defined the weight initialization and scoring of genes for each genomic profile. We then performed benchmarking of iDRW with four state-of-the-art pathway-based approaches (PLAGE, z-score, DART and DRW) by integrating gene expression and copy number alteration data and using a single genomic profile as a baseline for two different cancers. We show that the proposed method contributes to an improved survival prediction performance for both breast cancer and neuroblastoma datasets, despite heterogeneity in the data., We also jointly analyze multiple genomic profiles for two different cancer types in the integrated gene-gene graph by visualizing the gene-gene interaction network and identifying biologically meaningful pathways and genes. The overall process of the proposed framework is illustrated in Fig. 1. 

## Methods

### Dataset

mRNA expression data and copy number alterations data of breast cancer patients were obtained from the METABRIC dataset [20]. mRNA expression data were obtained as Illumina Human v3 microarrays with log-intensity levels. DNA copy number alterations were obtained and calls are made after normal contamination correction and copy number variation removal using thresholds. These values were: − 2 = homozygous deletion; − 1 = hemizygous deletion; 0 = neutral / no change; 1 = gain; and 2 = high level amplification. 24,368 and 22,544 genes of the gene expression data and the putative copy number alterations from the overlapping 1904 samples were used. 313 missing values of gene expression profiles and copy numbers data were imputed as the median of the corresponding patients’ data. The patients were categorized as having good (> 10 years) or poor (≤ 10 years) group with respect to their survival days. The cutoff of 10 years was arrived as being the median survival days of 1904 samples. We excluded 256 samples in which the survival was less than 10 years and reported as living. In total, 908 samples of the good and 740 samples of the poor group were used out of 1648 samples. We normalized the expression values of the mRNA gene expression data so that the mean was 0 and standard deviation was 1.

In the Neuroblastoma dataset, gene expression profile and copy number data were obtained from GSE49711 [21–23] from the GEO database [24]. Gene expression profiles of RNA sequencing for 60,586 genes and copy number data for 22,692 genes were obtained from the overlapping 144 samples. Raw microarray data from Microarray-based Comparative Genomic Hybridization (aCGH) were preprocessed using the rCGH R/Bioconductor package [25] with default parameter settings, using the circular binary segmentation algorithm (CBS) [26] and then converting into DNA copy number table over genes. The missing values for each profile were imputed using the median value of the corresponding patients’ data, and we excluded 331 genes which had more than half of the missing values. Finally, we divided 144 patients: 38 samples into the good group and 105 samples into the poor group, according to the predefined binary class label for overall survival days as provided by GSE62564 [27].

### Pathway-based integrated gene-gene graph construction

To integrate pathway information on multiple genomic profiles, we utilized an integrative directed random walk-based pathway activity inference method (iDRW) for two different types of omics data. To apply the iDRW method, we redesigned a directed gene-gene graph for gene expression and copy number data. We first collected the whole set of human pathways and the corresponding gene sets from the KEGG database [28]. Interactions between genes were defined in the gene-gene graph guided by the pathway information using the R KEGGgraph package [29]. Finally, an integrated directed gene-gene graph was constructed, consisting of 7390 nodes and 58,426 edges from 327 human pathways. Details about the construction method of the global directed graph are provided in [11]. To integrate gene expression and copy number alterations data on the graph, we included all edges within each gene expression profile. To consider the impact of copy number alteration on gene expression [30], we only assigned directional edges to the overlapping genes from copy number to gene expression data, and all edges between genes of copy number data were excluded. As two or more copies can be occurred in genes, directed edges can be assigned between genes in copy number alteration data and multiple genes in gene expression data in the integrated graph.

### Integrative directed random walk-based method (iDRW)

We applied the iDRW method to the pathway-based gene-gene interaction graph constructed from gene expression and copy number data. In the algorithm a random walk is performed on the integrated gene-gene graph for each cancer dataset. For each profile, the initial weight vector of the genes *W*_0_ was assigned as:

$$ {W}_0=-\mathit{\log}\left({w}_g+\epsilon \right) $$

$$ {W}_{t+1}=\left(1-r\right){M}^T{W}_t+r{W}_0 $$

where *w*_*g*_ is the weight of the gene *g* in the integrated gene-gene graph, and *ϵ* = 2.2*e*^−16^. As the iDRW method is specifically designed for gene expression profiles and methylation features, the weight initialization scheme was modified to reflect the distribution of each profile. For the breast cancer dataset, the weight of the gene is the *p*-value from either a two-tailed *t*-test for the mRNA expression profile or a *χ*^2^-test of independence for copy number genes. The *χ*^2^-test of independence was used as the copy numbers are discrete values. A *χ*^2^-test of independence is a nonparametric statistical test used to determine if the two or more classifications of the samples are independent or not, and can be applied only to discrete data [31]. In the neuroblastoma data, the *p*-value of RNA-Seq genes were measured by DESeq2, which is a state-of-the-art technique for the differential analysis of gene expression based on a negative binomial distribution for RNA-Seq data [32]. The weight vector for each gene is normalized to be between 0 and 1, and *W*_0_ is L1-normalized to a unit vector.

A random walker starts on a source node *s* and transits to a randomly selected neighbor or returns to the source node *s* with a restart probability *r* at each time step *t*. The weight vector *W*_*t*_ is iteratively updated at time step *t* and is guaranteed to converge to a steady state *W*_∞_ when ∣*W*_*t* + 1_ − *W*_*t*_ ∣  < 10^−10^, as shown in the DRW method [11]. *M* is a row-normalized adjacency matrix of the integrated gene-gene graph. We set the restart probability *r* to 0.7, which is default value of the DRW method, as it was previously shown that the performance of the DRW method is not sensitive to variations in *r* [11].

For a *j*-th pathway *P*_*j*_ containing *n*_*j*_ differential genes $\left({g}_1,{g}_2,\dots, {g}_{n_j}\right)$ whose *p*-value (*w*_*g*_) is < 0.05, the pathway activity is defined as:

$$ a\left({P}_j\right)=\frac{\sum \limits_{i=1}^{n_j}{W}_{\infty}\left({g}_i\right)\ast score\left({g}_i\right)\ast z\left({g}_i\right)}{\sqrt{\sum \limits_{i=1}^{n_j}{\left({W}_{\infty}\left({g}_i\right)\right)}^2}} $$

where *W*_∞_(*g*_*i*_) is the weight of gene *g*_*i*_ from the DRW method, *z*(*g*_*i*_) is the normalized expression vector of *g*_*i*_ across overall samples, and *score*(*g*_*i*_) is either a *log*_2_ *fold change* from the DESeq2 analysis for a RNA-Seq gene, or a *sign*(*tscore*(*g*_*i*_)) where *tscore*(*g*_*i*_) is a *t*-value from two-tailed *t*-test statistics for a mRNA expression gene. The sign of a *t*-value indicates the direction of the significant difference in sample group means. For copy number data, we scored each gene by *mean*(*CNA*(*g*_*i*_)_*poor*_) − *mean*(*CNA*(*g*_*i*_)_*good*_) where *CNA*(*g*_*i*_)_*poor*_ and *CNA*(*g*_*i*_)_*good*_ are the copy numbers of genes in the samples for the poor or good groups, reflecting the mean difference between the two groups. The *score*(*g*_*i*_) represents how much the values of gene *g*_*i*_ have changed between groups of samples. More details of the DRW method and the pathway activity inference method are provided in [9]. For each pathway, the pathway activity is computed from the gene expression and copy number values for each sample, which corresponds to a pathway profile.

### Pathway feature selection and survival prediction

To select important pathway features, 327 human pathways were ranked by their *p*-values from the *t*-test of pathway activities across samples. The top-*k* pathway features across samples were used as an input to a classification model. For each model, the hyper-parameter *k* was empirically set to the optimal one which shows the best classification performance with varying *k* between 5 and 50 in increments of five.

For the final survival prediction, a logistic regression model was applied. The regression model classifies the samples into either the good or the poor groups. The classification performances are measured using accuracy, precision, recall, and F-1 scores. Accuracy is a ratio of correctly predicted observation to the total observations. Precision is the ratio of correctly predicted positive observations to the total predicted positive observations. Recall (Sensitivity) is the ratio of correctly predicted positive observations to the all observations in actual class. F-1 Score is the weighted average of precision and recall. While accuracy intuitively measures how correctly the model classifies the samples into two survival groups, precision or recall takes the costs of false positives and false negatives into account. When class distribution is uneven, the performance of classification model should be measured with precision and recall. In our experiments, we used accuracy as a performance measure, and we also validated with precision, recall and F1-score for further experiments.

### Performance evaluation

In breast cancer data, we evaluated the classification performance with 5-fold cross-validation. We divided the whole samples into five folds. The classification model was trained using four folds and validated using the remaining fold. The entire process was repeated 50 times and then we assessed the accuracy, precision, recall and F-1 score after the entire 250 iterations, using the selected top-*k* pathway features as a final classification performance. As the number of samples in neuroblastoma data is imbalanced and not enough to perform 5-fold cross-validation, we evaluated the classification performance using a leave-one-out cross-validation, which leaves one sample as a validation set and trains a classification model with remaining samples for each iteration.

To investigate the utility of the iDRW method using breast cancer and neuroblastoma datasets, we compared iDRW with four state-of-the-art pathway-based methods: PLAGE, z-score, DART, and DRW. PLAGE and the z-score method were implemented using the R GSVA package with default settings [33]. In the experiments, the pathway activity scores across samples were obtained from the gene expression data using the four pathway-based methods. In order to ensure a fair comparison, the top-*k* pathway features selection and the classification performance evaluation of the four pathway-based methods and the iDRW method were conducted as stated above. As a baseline, we evaluated the classification performance with single gene expression profile. The top-*k* genes which are ranked by *w*_*g*_ which are their *p*-values from a statistical test were used to train the classification model.

To demonstrate the robustness of the proposed model, the models are tested with different hyper-parameter value settings for *k*. We also assessed classification performances for varying training data size. In this experiment, the parameter *k* is set to the optimal one for each method.

## Results

### iDRW improves survival prediction performance compared to other pathway-based approaches

**Fig. 2:**
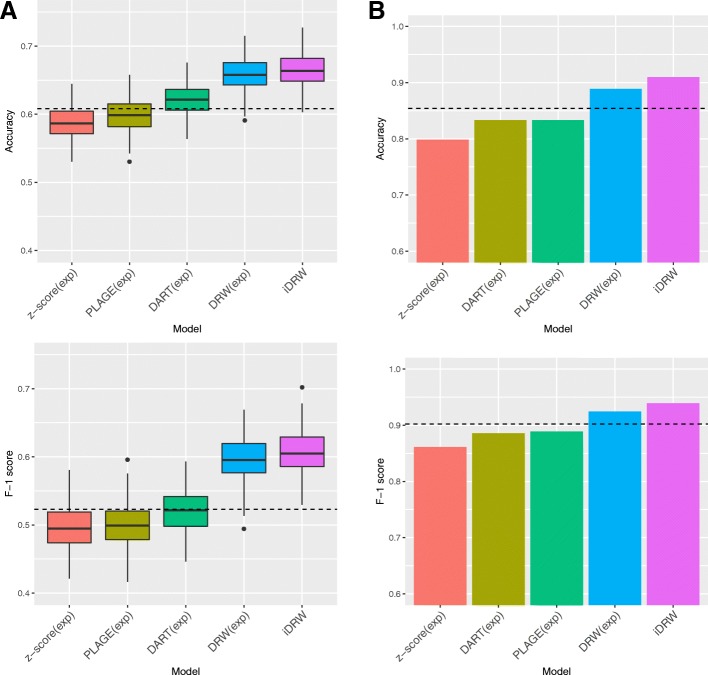
Survival prediction performance comparison between pathway profiles of four pathway-based methods on the gene expression data and those of the iDRW method on the gene expression and copy number data in breast cancer (**a**) and in neuroblastoma data (**b**). Performance is measured with accuracies and F-1 scores after 50 repeats of five-fold cross-validation with top-k pathways (**a**). In the neuroblastoma data, performances are measured using leave-one-out cross-validation due to the sample size (**b**). The value of *k* is empirically set to the optimal one for each method. The performance of the gene expression profile is shown as a dotted horizontal line

We assessed the survival prediction performances using four pathway-based methods with a single gene expression profile and the iDRW method on the gene expression profile and copy number data both in breast cancer and neuroblastoma patients. Figure 2a shows the prediction performances after 50 repeats of 5-fold cross-validation. Performances were measured using accuracy and F-1 score. We note that the classification performances of neuroblastoma data were evaluated with leave-one-out cross-validation since the sample size is extremely small, as shown in Fig. 2b. We compared the classification performances of the iDRW method with four state-of-the-art pathway-based approaches: PLAGE, z-score, DART and the DRW method. The four pathway-based approaches are implemented using gene expression profiles only, and the iDRW method is performed on the combined gene expression and copy number data. As a baseline, the classification performance of a single gene expression profile is shown as a dotted horizontal line. We used the top-*k* pathway features across samples as an input to the classification model, and the optimal value of *k* is set to that which shows the best classification accuracy for each method. The optimal parameter *k* for each model is denoted at x-axis label. When single gene expression profile was used, the top-50 gene features in breast cancer data or top-10 genes in neuroblastoma data were used.

Although the performances of the z-score and PLAGE were worse than those of the gene expression profile, the performances were improved when DART, DRW and iDRW utilizing the pathway information were used. In particular, DRW-based methods contribute to an enhance classification performance in both cancer datasets investigated. This research demonstrates that DRW-based approaches which utilizes topological information of genes on a pathway-based integrated graph is a more effective way of inferring pathway activities than other methods. The iDRW method on the combined feature data performed the best amongst all of the other methods used despite the heterogeneity in gene expression and copy number data. These results demonstrate that the iDRW method successfully represented the combined effects of multiple genomic profiles on a pathway-based integrated graph both in breast cancer and neuroblastoma data.

**Fig. 3:**
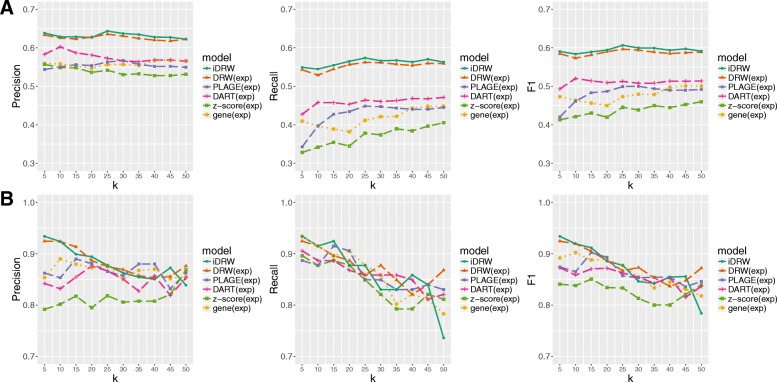
Classification performances of the iDRW method and four pathway-based methods with varying values of *k* for breast cancer (**a**) and neuroblastoma data (**b**). Classification performances with top-*k* pathway features are shown for each model with varying *k* = 5, 10, …, 45, 50. Performance is measured using precision, recall and F-1 score after 50 repeats of five-fold cross-validation in breast cancer data (**a**) and leave-one-out cross-validation in neuroblastoma data (**b**)

We evaluated the classification performances with top-*k* pathway features for each model with values of *k* varying from 5 to 50 in increments of five with respect to precision, recall and F-1 score for breast cancer data (Fig. 3a) and for neuroblastoma data (Fig. 3b). In breast cancer data, we observe that DRW-based approaches show higher accuracy and more stable performance with respect to the change in *k*. DART infers pathway activities using genes encoding hubs in expression correlation networks and shows better performances than other benchmark pathway-based approaches. As z-score and PLAGE measure pathway activity profiles by summarizing scores of gene sets for each pathway, prediction performances tend to increase with more number of features. When taking both precision and recall into account, the results revealed that DRW-based pathway activity profiles lead to a more stable performance and less sensitive result to the number of features compared to other pathway-based approaches. It indicates that top-ranked 10 to 25 pathways and corresponding significant genes obtained from DRW-based pathway activity profiles represent meaningful markers enough to train the classification model. In case of neuroblastoma dataset (Fig. 3b), the performances of the all the methods are more sensitive to the change of *k*. This appears to be because the neuroblastoma dataset is relatively small and hence it becomes more critical to choose the optimal hyper-parameter value. It is observed that around the optimal values of *k*, the performances of DRW-based methods are substantially better than the others.

**Fig. 4:**
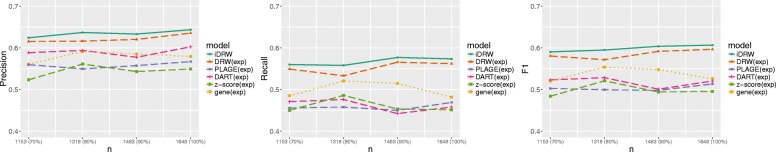
Classification performances of the iDRW method and four pathway-based methods with varying number of sample size N in breast cancer samples. Classification performances are shown with respect to the number of samples N which are 70, 80, 90, 100% out of whole samples. Performances are measured using precision, recall and F-1 score after 50 repeats of five-fold cross-validation in breast cancer data

Figure 4 shows the performance behavior with respect to the data size variation by using 70 to 100% of the entire samples in the experiments. For example, when 70% of samples in breast cancer dataset were used, we randomly sampled 1153 out of 1648 samples, which are then used for 5-fold cross validation. The experiments are performed only with breast cancer data due to extremely small size of the neuroblastoma dataset. The neuroblastoma dataset has 144 samples as a whole and further reduction in the training data size may not lead to meaningful performance results. And the actual difference in the number of used samples in 70 and 100% setting is also small, so we only experimented with breast cancer samples.

Performances were measured in terms of precision, recall and F-1 score with the optimal value of *k*. We observe that the performances of DRW-based approaches were superior to other pathway-based approaches and single gene expression profile by showing that those of DRW and iDRW showed more stable and better performances across varying data sizes. When the recall is considered, the performances of pathway profiles obtained from z-score, PLAGE and DART were worse than the one from single gene expression profile. As the genes in gene expression profile were weighted by their statistical significance values and top-*k* genes were selected that yielded the best classification accuracy, it seems to contribute to the higher performance than other approaches of z-score, PLAGE and DART which don’t use statistical difference information of two survival groups.

### iDRW identifies cancer-associated pathways and genes

The iDRW method has the advantage in that we can jointly identify genes which are differentially expressed or have differential changes in copy number in the top-ranked pathway features. Table 1 shows the selected top-*k* pathways and corresponding gene sets ranked by the iDRW method from gene expression and copy number data in breast cancer (*k* = 25) and neuroblastoma data (*k* = 5). The total number of genes and significant genes from the gene expression and copy number data are shown for each pathway. The significant genes from the gene expression and copy number data are those genes whose *p*-value of a significant test is lower than 0.05.

**Table 1:** Top-*k* pathways ranked by the iDRW method in breast cancer (*k* = 25) and neuroblastoma data (*k* = 5). For each pathway, total number of genes, significant genes from gene expression (EXP) and copy number data (CNA) are shown (*p*-value of *t*-test / DESeq2 or ***χ***^**2**^-test < 0.05)

Dataset	Pathway ID	Pathway name	Total genes	EXP	CNA
Breast cancer	hsa04740	Olfactory transduction	419	54	268
	hsa04014	Ras signaling pathway	232	68	164
	hsa04015	Rap1 signaling pathway	206	64	142
	hsa04916	Melanogenesis	101	37	73
	hsa04722	Neurotrophin signaling pathway	119	38	84
	hsa05200	Pathways in cancer	526	166	359
	hsa04933	AGE-RAGE signaling pathway in diabetic complications	99	37	67
	hsa04530	Tight junction	170	53	107
	hsa04510	Focal adhesion	199	76	125
	hsa04080	Neuroactive ligand-receptor interaction	278	64	193
	hsa05225	Hepatocellular carcinoma	168	56	112
	hsa04020	Calcium signaling pathway	182	59	136
	hsa04024	cAMP signaling pathway	198	58	139
	hsa04217	Necroptosis	164	49	97
	hsa04060	Cytokine-cytokine receptor interaction	270	70	192
	hsa05152	Tuberculosis	179	58	112
	hsa05165	Human papillomavirus infection	319	103	210
	hsa04810	Regulation of actin cytoskeleton	208	64	132
	hsa04151	PI3K-Akt signaling pathway	352	119	241
	hsa04022	cGMP-PKG signaling pathway	163	58	109
	hsa04630	Jak-STAT signaling pathway	162	43	112
	hsa05167	Kaposi’s sarcoma-associated herpesvirus infection	186	61	114
	hsa04010	MAPK signaling pathway	295	87	209
	hsa04371	Apelin signaling pathway	137	46	99
	hsa04390	Hippo signaling pathway	154	58	100
Neuroblastoma	hsa04976	Bile secretion	71	13	5
	hsa05034	Alcoholism	180	22	7
	hsa01100	Metabolic pathways	1273	43	93
	hsa04080	Neuroactive ligand-receptor interaction	278	21	24
	hsa04151	PI3K-Akt signaling pathway	352	19	31

Hanahan and Weinberg have established six biological capabilities which are acquired during tumor generation: sustaining proliferative signaling; evading growth suppressors; activating invasion and metastasis; enabling replicative immortality; inducing angiogenesis; and resisting cell death [34]. We found that some of the top-25 ranked pathways in breast cancer data are related to at least one of six functions, such as the Ras signaling pathway (KEGG ID: hsa04740), Necroptosis (KEGG ID: hsa04217), Regulation of actin cytoskeleton (KEGG ID: hsa04810), and the PI3K-Akt signaling pathway (KEGG ID: hsa04151) [34]. Olfactory receptors are known to act on cell migration, proliferation, and secretion in a variety of human tissues, and function as biomarkers for breast cancer [35], which indicates a relationship between the top-ranked pathway, the olfactory transduction pathway (KEGG ID: hsa04740) and breast cancer. In particular, the expression of Olfactory Receptor Family 2 Subfamily B Member 6 (OR2B6), which is a differentially expressed gene, was detected in most breast carcinoma tissues [36]. The development of cancer is closely linked to viral infection, and breast cancer is known to be associated with viruses of the herpesvirus, polyomavirus, and retrovirus families [37]. This information indicates that Human papillomavirus infection (KEGG ID: hsa05165) and Kaposi’s sarcoma-associated herpesvirus infection (KEGG ID: hsa05167) are related to breast cancer [38]. Thus, we assume that the top-ranked pathways can play a crucial role on breast cancer mechanism and differentiate survival groups of patients.

The top five pathways involved in neuroblastoma were identified using the iDRW method. Several previous studies suggested that top five pathways in Table 1 are relevant to neuroblastoma. For example, an in vitro research project suggested a mechanism underlying a potent and selective anti-tumor effect of lithocholic bile acid in neuroblastoma cells [39], which shows the relation between the bile secretion pathway (KEGG ID: hsa04976) and the neuroblastoma. Alcoholism pathway (KEGG ID: hsa05034) includes the reaction to ethanol in a dopaminergic neuron [40]. Several studies have shown that the level of Urinary catecholamine metabolites including vanillylmandelic acid (VMA), homovanillic acid (HVA) and dopamine are elevated in neuroblastoma patients [41, 42]. Furthermore, the neuroactive ligand-receptor interaction pathway (KEGG ID: hsa04080) and metabolic pathways (KEGG ID: hsa01100) are associated with neuroblastoma, since neuroblastoma occurs in nerve tissue and changes in metabolism are common phenomena in cancer [34]. We found some evidences that the PI3K-Akt signaling pathway (KEGG ID: hsa04151) aids in the pro-survival of neuroblastoma [43–45]. Based on these findings, we hypothesized that the top five pathways can be associated with neuroblastoma and can be crucial features for distinguishing between two survival groups.

### The pathways and genes are jointly analyzed in the gene-gene network

**Fig. 5:**
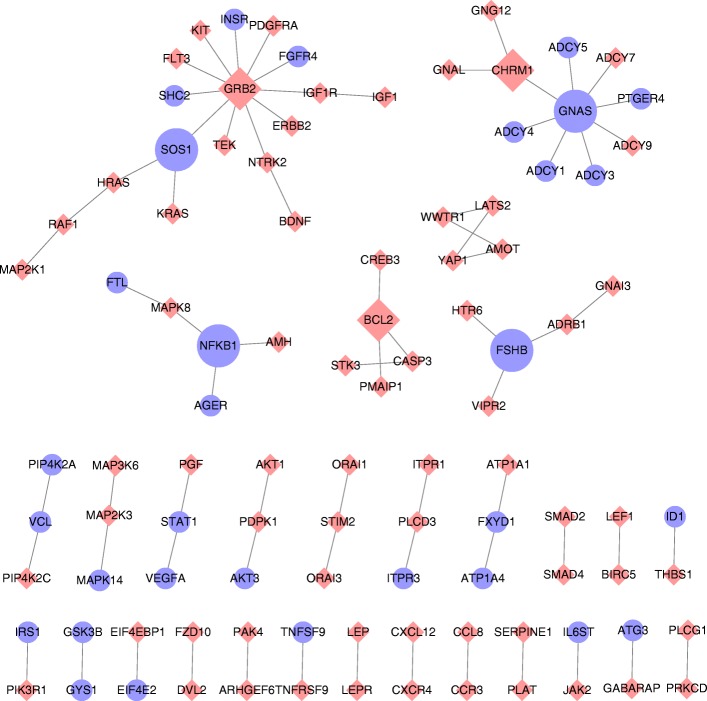
Pathway-based gene-gene interaction network between gene expression profile and copy number data in breast cancer samples. The genes in the top-25 pathways ranked by the iDRW method in the breast cancer data are shown. The hub genes whose degree is equal to or greater than three in the gene expression profile (blue ellipses) and genes in copy number data (pink diamonds) are emphasized in the network

The interactions between significant genes in the top-25 pathways in the breast cancer dataset (Table 1) are visualized in the gene-gene network shown in Fig. 5. The gene-gene network of neuroblastoma is not shown, as the number of edges between genes in the top five pathways were few. The hub genes whose degree in the network is equal or greater than three play a crucial role in pathways selected by the iDRW method. Several studies have identified relationships between the survival of breast cancer patients and the hub genes in the network: specifically the GNAS complex locus (GNAS), growth factor receptor bound protein 2 (GRB2), follicle stimulating Hormone Subunit Beta (FSHB), Cholinergic Receptor Muscarinic 1 (CHRM1), SOS Ras/Rac Guanine Nucleotide Exchange Factor 1 (SOS1), Nuclear Factor Kappa B Subunit 1 (NFKB1), and the BCL2 Apoptosis Regulator (BCL2). It has been reported that the amplification of GNAS may contribute to the pathogenesis of breast cancer and is associated with the survival of patients with invasive breast carcinoma [46, 47]. In addition, GRB2 and SOS1 have been reported to be overexpressed in breast cancer tissues compared with normal tissues [48, 49].

## Discussions

In this study, we demonstrated the effectiveness of DRW-based approaches and the interaction effects between multiple genomic profiles on the integrated graph. However, the results in neuroblastoma samples showed a different tendency and didn’t confirm clear performance improvement of DRW-based approaches with increasing *k*. We found that statistically significant expressed genes in neuroblastoma data are relatively small compared to in copy number alterations data and those in breast cancer data as shown in Table 1. iDRW performed random walk process on the integrated graph which reflects the impact of copy number genes on gene expression and DRW reflected interactions between genes from gene expression data. Other pathway-based approaches: z-score, PLAGE and DART were implemented using gene expression profile. Therefore, there is a possibility that the effect of gene expression profile and the impact of copy number alterations on gene expression on the graph can be undermined. As the clear tendency was not shown due to the extremely small sample size in neuroblastoma data, we can reduce the limitations when more samples were obtained or other genomic profiles can be utilized as well as gene expression and copy number alterations data. In future works, the clear criteria of dividing two survival groups are needed or it can be extended to the survival analysis by training regression model which predicts the actual survival days of patient samples.

## Conclusions

In this study, the effectiveness of a directed random walk-based multi-omics data integration method was investigated and analyzed using datasets incorporating gene expression and copy number alterations for two different cancer datasets. To integrate the gene expression and copy number alteration data, we first constructed a directed gene-gene graph representing the impact of copy number variants on gene expression by defining weight initializations and gene scoring measures for each genomic profile. To demonstrate the utility of the iDRW method, the performances of four state-of-the-art pathway-based methods: PLAGE, z-score, DART and DRW were compared with the survival prediction model which classifies samples into two survival groups. The results demonstrate that the iDRW method, which utilizes the interactions between genes on an integrated gene-gene graph, produced the best classification performance for both breast cancer and neuroblastoma data. It shows that the integrated gene-gene graph successfully reflected the combined effect of gene expression and copy number alterations data, guided by pathway information for both cancer datasets. From a joint analysis of multiple genomic profiles, the iDRW method can identify biologically meaningful pathways and genes highly related to the cancer under investigation. We also visualized the gene-gene interactions between gene expression, and copy number alterations data in the integrated gene-gene graph for both the breast cancer and neuroblastoma datasets.

## Reviewers’ comments

### Reviewer’s report 1: Helena Molina-Abril

**Reviewer summary**

In this paper the authors apply their previously developed method iDRW on multiple genomic profiles for two different cancers and redesign a directed gene gene graph which reflects the interaction between gene expression and copy number data. They also compare the iDRW method with other pathway-based methods for a survival prediction model which classifies samples into two survival groups.

**Reviewer recommendations to authors**

In my opinion the paper needs some language corrections (english quality) as well as some methodological corrections before being published.

**Author’s response:** *We have carefully proofread our manuscript and revised grammatical errors and unclear sentences.*

Further explanations of some concrete points need to be addressed. Data sources as well as preprocessing is too briefly explained. For instance, mRNA data normalization and the imputation method for the neuroblastoma dataset is not mentioned.

**Author’s response:** *We have added a more detailed description of METABRIC breast cancer dataset and neuroblastoma dataset in the GEO database provided by CAMDA 2018 data integration challenge in “Dataset” section of “Methods”. Explanations of data preprocessing such as mRNA data normalization, aCGH microarray raw data processing, and median imputation method for the neuroblastoma data are also included.*

The generation of an integrated directed gene-gene graph is vaguely described as well as the KEGG pathway selection (327?). Authors should give a clearer explanation of this process.

**Author’s response:** *We have added a new sub-section “Pathway-based integrated gene-gene graph construction” in “Methods” to provide a more detailed and clearer explanation of how the integrated graph is constructed to reflect the interactions between gene expression and copy number alterations. As the total number of human pathways we obtained in KEGG database is 327, we also revised the description of how the pathways and corresponding gene sets were collected more clearly.*

The use of t-test of chisquared test values for initial weights is not sufficiently reasoned.

**Author’s response:** *We used two tailed t-test for mRNA expression data, chi-squared test of independence for copy number data, and DESeq2 measure for RNA-Seq data to consider the different data distributions. We added clear explanations of each statistical test and weight initialization of genes with equations in section “Integrative directed random walk-based method (iDRW)” in “Methods”. We also added more explanations of gene scoring measures for each genomic profile when pathway activity inference is performed.*

Accuracy is not an appropriate performance measure for imbalanced data sets. Please change that.

**Author’s response:** *Considering the reviewer’s valuable suggestion, we evaluated the performance with precision, recall and F-1 score in addition to accuracy, and added a description of each performance measure in section “Pathway feature selection and survival prediction” of “Methods”. In* Fig. 2*, the classification performances were evaluated in terms of accuracy and F-1 score for both cancer datasets. In addition, we performed additional experiments to investigate the performance behavior with respect to changes in the parameter k (*Fig. 3*) and the number of samples (*Fig. 4*) using precision, recall and F-1 score.*

I’m also concerned about the logistic regression model. No testing data has been used for validation, and therefore classification results might be too optimistic.

**Author’s response:** *To evaluate the classification performance in breast cancer data as an example, we have performed 5-fold cross-validation which divided the whole samples into five folds and used four folds as training data and the remaining fold as validation data. We repeated the entire 5-fold cross-validation process 50 times and obtained the average performance of 250 iterations as a final classification performance. The descriptions of cross-validation process are described in the first paragraph of section “classification performance evaluation” in “Methods”.*

Feature selection, does not seem to be included within the learning process, which may lead to biased results.

**Author’s response:** *For feature selection, we first ranked pathway features using their statistical significance and then determined the optimal number of top-k ranked feature set which yields the best cross validation accuracy. For a clear explanation of this process, we revised the first paragraph of section “Pathway feature selection and survival prediction” and “Performance evaluation” in “Methods”.*

In general, the paper is based on a previously published method, but applied to a new dataset. It is not sufficiently clear what is its substantial contribution and novelty.

**Author’s response:** *We clarified the main contributions of this study to differentiate our method from the previously published method in the last paragraph of “Background” and in “Conclusions”. In the current paper, we proposed an integrated gene-gene graph construction method reflecting interactions between copy number alterations and gene expression data for two different cancer types. We also demonstrated improved prediction performance by a comparative analysis of iDRW with four state-of-the-art pathway-based approaches. By visualizing the gene-gene interaction network on the combined profiles, we could jointly analyze multiple genomic profiles on the integrated gene-gene graph, and we could also identify biologically meaningful pathways and genes.*

Abbreviations should be first mentioned with its corresponding name (see for instance Array comparative genomic hybridization (aCGH)

**Author’s response:** *We added the full name of all abbreviations including aCGH throughout the entire manuscript and we also listed them in “List of abbreviations”.*

### Reviewer’s report 2: Marta Hidalgo

**Reviewer summary**

Integration of different types of genomic data is a major open problem. This paper presents a new method for survival prediction through the integration of gene expression and copy number data in a pathway model. It also presents the comparison of the performance of the described model with other 4 pathways methods in terms of prediction of survival groups. In general the paper is well written, although some paragraphs and sentences are somehow not clear enough. In particular, it should be explained with more detail how the integration is performed. Also some language improvements should be addressed before publication.

**Reviewer recommendations to authors**

MINOR RECOMMENDATIONS: The major concern that I find is that although integration is one of the key points of the method, called iDRW, and the one new feature with respect to the DRW method on which it is based, the explanation of how this integration is performed is not clear enough.

**Author’s response:** *As the reviewer pointed out, the integration method of gene expression copy number data based on a gene-gene graph guided by the pathway information is one of the main contributions. We have included a detailed and clearer explanation of how the integrated graph is constructed to reflect the interactions between gene expression and copy number alterations data in a new sub-section “Pathway-based integrated gene-gene graph construction” in “Methods”.*

Also, I would appreciate a mention to the kind of data used by the other methods: are they also accepting both gene expression and copy number data? If not, then an explanation of why these methods are appropriate to be compared with iDRW should be given.

**Author’s response:** *The existing pathway activity inference methods focused on how to effectively incorporate pathway information into genomic analysis and they were implemented to handle only single genomic profile. In this respect, the iDRW method has been proposed for combining multiple genomic profiles on an integrated gene-gene graph constructed by pathway information. Therefore, we used gene expression profile for four pathway-based methods: z-score, PLAGE, DART and DRW method, and gene expression and copy number data for iDRW method in this study. We added further explanations of each pathway-based method and iDRW method in “Background”. We stated that only iDRW method were implemented by incorporating gene expression and copy number data in section “iDRW improves survival prediction performance compared to other pathway-based approaches” in “Results”.*

Sentences to be revised:

- Fourth sentence in the last paragraph of “Background”.

- Second sentence in first paragraph of section “Results and discussion”, subsection “Integrative analysis...”.

- First sentence in first paragraph of section “Results and discussion”, subsection “iDRW identifies...”.

Spelling typos:

- When defining the initial weights, after W_0 should say “are”.

- In sign (tscore(g_i)), should it be sign (score(g_i))?

- Before “More details of the DRW method...” should be a “.”.

- First sentence in first paragraph of section “Results and discussion”, subsection “iDRW identifies...”, “differential” should be “differentially”.

- Second sentence of second paragraph in the same section, “some of top-25” should be “some of the top-25”.

- Last sentence in the same paragraph, “crucial role of breast cancer” should be “crucial role on breast cancer”.

- Fifth sentence in next paragraph, “and dopamine elevated” should be “and dopamine are elevated”.

**Author’s response:** *We revised all the unclear sentences as the reviewer suggested and thoroughly proofread the entire manuscript. We appreciate the reviewer’s kind corrections and suggestions.*

## Abbreviations

aCGH: Microarray-based comparative genomic hybridization
CBS: Circular binary segmentation algorithm
CNVs: Copy number variants
DRW: Directed random walk
